# Near-Infrared Spectroscopic Determination of Pentacyclic Triterpenoid Concentrations in Additives for Animal Food

**DOI:** 10.3390/biology13080578

**Published:** 2024-07-31

**Authors:** Carmen Sugráñez-Pérez, Rafael Sugráñez-Serrano, Marta López-González, Sara Martínez-Vaquero, Daniel Moral-Martos, Sofía Cortés-Jiménez, Juan Peragón-Sánchez

**Affiliations:** 1 Agropecuaria Loma de Los Donceles (Agroloma), 23400 Úbeda, Jaén, Spain; carmensugranez@agroloma.com (C.S.-P.); rafaelsugranez@agroloma.com (R.S.-S.); 2 CCPA Group, Nutega S.L., 28823 Madrid, Spain; mlopez@nutega.com (M.L.-G.); smartinez@nutega.com (S.M.-V.); 3 Biochemistry and Molecular Biology Section, Department of Experimental Biology, University of Jaén, 23071 Jaén, Spain; dmm00038@red.ujaen.es (D.M.-M.); scj00008@red.ujaen.es (S.C.-J.)

**Keywords:** maslinic acid, oleanolic acid, uvaol, near-infrared spectroscopy, oxygen radical absorbance capacity

## Abstract

**Simple Summary:**

Olive tree and plant by-products can be used for enhance the nutritional composition of food due to their high content of bioactive compounds such as pentacyclic triterpenes. Here, we present a novel application of near-infrared spectroscopy for the prediction of the total or individual (maslinic acid, oleanolic acid, and uvaol) pentacyclic triterpene concentrations in a feed additive obtained from a plant mixture. This method can be applied directly to dried powder and makes extraction and high-performance liquid chromatography analysis unnecessary. It can be used at the factory level to directly determine the pentacyclic triterpene concentrations in the additive powder at the same time that the powder is produced.

**Abstract:**

The nutritional composition of food for animal production can be enhanced using olive tree and plant by-products due to their high content of bioactive compounds such as pentacyclic triterpenes. Here, we present a novel application of near-infrared spectroscopy (NIRS) for the prediction of the total or individual [maslinic acid (MA), oleanolic acid (OA), and uvaol (UO)] pentacyclic triterpene concentrations in a feed additive obtained from a plant mixture. The oxygen radical absorbance capacity of these types of samples demonstrated the existence of a high antioxidant capacity. The conventional determination methods of pentacyclic triterpene concentration are costly, labor-intensive, and not practical for analyzing several lines within a limited timeframe at the factory level. The optimal regression model developed in our work demonstrated high correlation values for the calibration and validation sets, along with a high residual prediction deviation value. We used 63 samples for the development of the model. The NIRS method can be applied directly to dried powder and makes extraction and high-performance liquid chromatography (HPLC) analysis unnecessary. Our results also demonstrate that NIRS can accurately quantify pentacyclic triterpenes even at low concentrations in food additives. It can be used at the factory level to directly determine the pentacyclic triterpene concentrations in the additive powder at the same time that the powder is produced.

## 1. Introduction

Pentacyclic triterpenes are secondary metabolites widely distributed in plants and have a nutraceutical role and important biological properties related to health and disease prevention [1]. They are found in high concentrations in *Olea europaea* organs, such as the fruits, leaves, stems, roots, and seeds [2,3]. Maslinic acid (MA), oleanolic acid (OA), ursolic acid (UA), betulinic acid (BA), erythrodiol (EO), and uvaol (UO) are the main pentacyclic triterpenoids found in this species [2,4]. In addition to health properties, several works have demonstrated that MA can be used as a feed additive in fish food [5,6,7,8]. The dietary addition of this compound produces increases in the growth and protein turnover rates in the liver and white muscle of trout and gilthead sea bream cultured in the laboratory or under farm conditions. In general, supported by the lack of adverse effects, the results obtained with purified molecules led us to consider these compounds as nutraceuticals [1].

The use of feed additives based on olive by-products has been the aim of many companies producing animal meat as a form of natural supplementation of pentacyclic triterpenoids. When the triterpenes studied are used as part of the plant matrix of the substrates that contain them (leaves, stems, roots, and seeds) instead of pure molecules, their concentrations are highly variable depending on the biological conditions and environmental challenges to which the plants are subjected. Therefore, the detection and quantification of the different triterpene content in each of the plant substrates used is necessary to formulate mixtures with the desired content. Having this information to formulate mixtures and confirm their efficacy once they are prepared is a requirement for the study and evaluation of triterpenes as an additive in food for animal production. The traditional method for triterpene quantification involves laboratory work, including extraction and analysis using ultraviolet (UV) or mass (MS) high-performance liquid chromatography analysis (HPLC) of the extracted samples. These are relatively complex laboratory techniques that need several days for their application. Near-infrared spectroscopy (NIRS) is a non-destructive method that is easy to use and can be an alternative to the traditional method of triterpene quantification. This methodology has several applications in the agricultural and olive sectors [9] related to quality control [10] and nutrient analysis [11,12]. In this context, an easy method to determine the concentrations of these compounds in the feed additives at the factory level while they are being produced is essential for these companies. Therefore, the aim of this study was the application of NIRS for the prediction of the concentrations of total or individual triterpenoids in feed additives obtained from a mixture of olive by-products and other plants. The samples used as feed additives were analyzed using HPLC-UV–Vis to identify and determine the concentrations of these compounds according to previously published works [2,3,13]. The same samples were analyzed using NIRS and the obtained NIRS spectra. The NIRS spectra are related to the pentacyclic triterpenoid concentrations, and an NIRS equation for the estimation of each triterpenoid was developed. Previously, the oxygen radical absorbance capacity (ORAC) was determined to measure the antioxidant capacity of these samples.

Previously, regression models based on NIRS spectroscopy were developed for the rapid determination of phenolic compound concentrations in several types of samples [14]. In this previous report, the authors described how the NIRS methodology is a more convenient method that can be used for phenols present in whole wheat products in industrial applications. NIRS methods are more effective than other methods for screening purposes when a large number of samples need to be analyzed.

To our knowledge, the study described in this work is the first study that directly used olive by-products to develop and report an NIRS method that effectively predicts the total or individual pentacyclic triterpenoid content in feed additives. This can have many practical applications for animal production or other advantages due to olive by-products.

## 2. Materials and Methods

### 2.1. Food Additive Samples and Chemicals

Plant samples from the Mediterranean ecosystem with industrial availability and abundant cultural and scientific information regarding their biological action in animal production were used. The tested product was a mixture of four plant samples from the leaves and seeds of lentiscus (*Pistacia lentiscus*), wild olive (*Olea europaea*), winemaking derivatives (*Vitis vinifera*), and thyme (*Thymus vulgaris*). After a selection and drying process at a low temperature, the different samples were weighed according to their formulation in batches of 500 kg. They were subsequently mixed, ground, and micronized with an air classifier mill, and a median granule lower than 400 μm and a particle size (geometric mean diameter—GMD) lower than 200 μm were obtained. Three identified 250 g samples were taken from each batch: one for shipment to Nutega S.L. (CCPA Group, Madrid, Spain) for the NIRS study, another to the University of Jaén for analysis of the triterpenoid concentration, and the third to the Agroloma (Agropecuaria Loma de Los Donceles, Úbeda, Jaén, Spain) sample library.

### 2.2. Extraction of Triterpenoids

We followed the procedure described in [2] for extraction and analysis of the triterpene’s compounds in the samples. Initially, the water content of 5 samples was determined by weighing 3 g of pulverized powder and drying this in an oven at 55 °C for 2 days until a constant weight was reached. After cooling, the samples were reweighed. The water content was 3.31 ± 0.02%, so the following analysis was conducted on wet samples.

For each sample, for the extraction of the triterpenoids, 0.125 g of dried powder was mixed with 1.5 mL of methanol/ethanol (1:1, *v*/*v*) and vigorously vortexed for 1 min. The samples were centrifuged at 7700× *g* for 5 min at 4 °C. The supernatants were collected, and the residue was re-extracted three times with the same volume of methanol/ethanol. All supernatants obtained after three extractions were mixed and evaporated using a SpeedVac. The residue was dissolved with 1 mL of methanol. These methanolic samples were filtered, and the analysis of the triterpenoids was carried out using an HPLC-UV–Vis chromatographic system.

### 2.3. Analysis and Determination of the Triterpenoid Content

**Figure 1:**
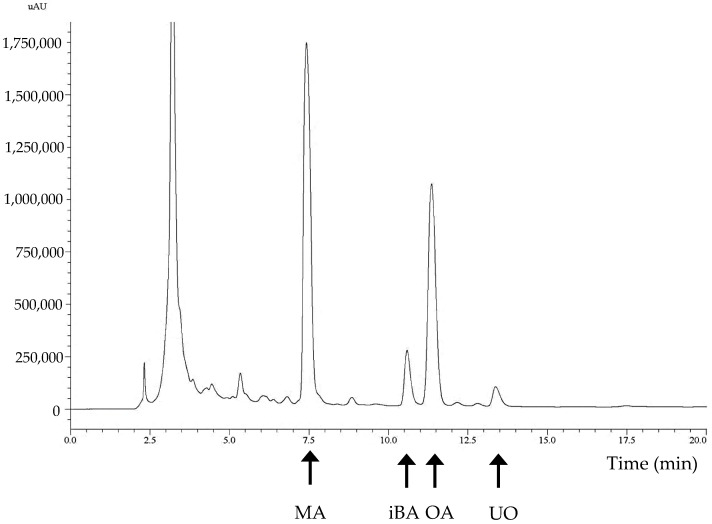
HPLC-UV–Vis chromatogram of plant additive. Twenty microliters of sample was analyzed using HPLC with a Spherisorb ODS-2 column that was eluted with methanol/water. The y-axis shows the absorbance at 210 nm. MA: maslinic acid; OA: oleanolic acid; UO: uvaol; iBA: betulinic acid isomer.

Reverse-phase chromatography was applied using a Spherisorb ODS-2 (Waters Corporation, Milford, CT, USA) column (2.5–4.6 mm, 5 µm). A Shimadzu HPLC system including two pumps, a column heater module, and a UV–Vis detector was used and operated with LC-Solutions software 5.42 SP3 (Shimadzu Corporation, Kyoto, Japan). An isocratic elution was applied using methanol/water with acetic acid (pH = 3.1) (92:8, *v*/*v*) for 20 min, with a flow rate of 0.8 mL min^−1^. The absorbance at 210 nm was recorded during elution. The chromatogram for the mixture is shown in Figure 1. The triterpenoids were identified and quantified using the external standard method, as indicated in [2,3]. Table 1 shows the average concentrations of these compounds found in all the experimental samples.

### 2.4. Collection of NIR Spectra, NIRS Model, and Equation Development

**Figure 2:**
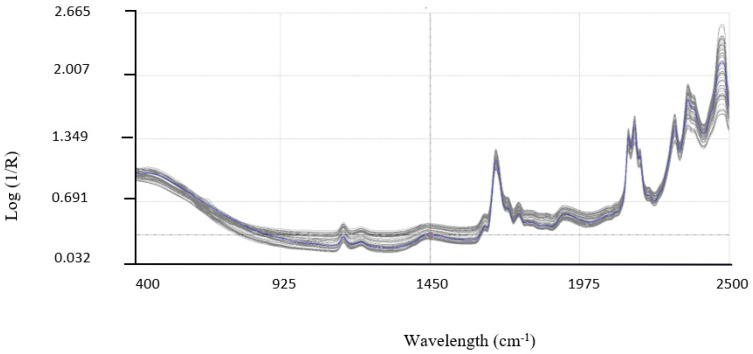
NIR spectra of plant additive samples ranging from 400 to 2500 cm^−1^ at a resolution of 2 cm^−1^. Color line indicates the most representative spectra.

**Figure 3:**
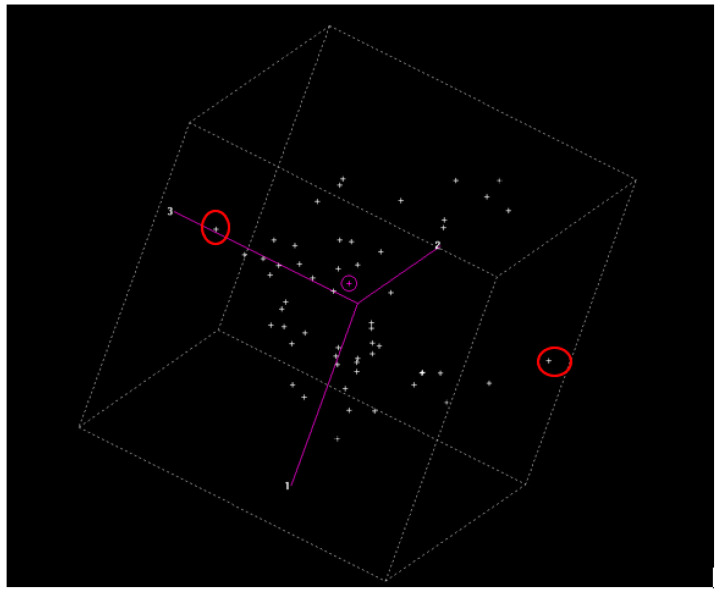
Principal component analysis of the complete spectra. Points circled in red correspond to the outlier’s samples.

All the samples were analyzed in triplicate from 400 cm^−1^ to 2500 cm^−1^, with increases of 2 cm^−1^, using NIR DS2500 equipment. The spectra obtained are plotted in Figure 2. The concentrations of pentacyclic triterpenes in the samples used for the development of the NIRS model are shown in Table 2. Figure 3 shows the intervals at which each parameter was distributed.

**Figure 4:**
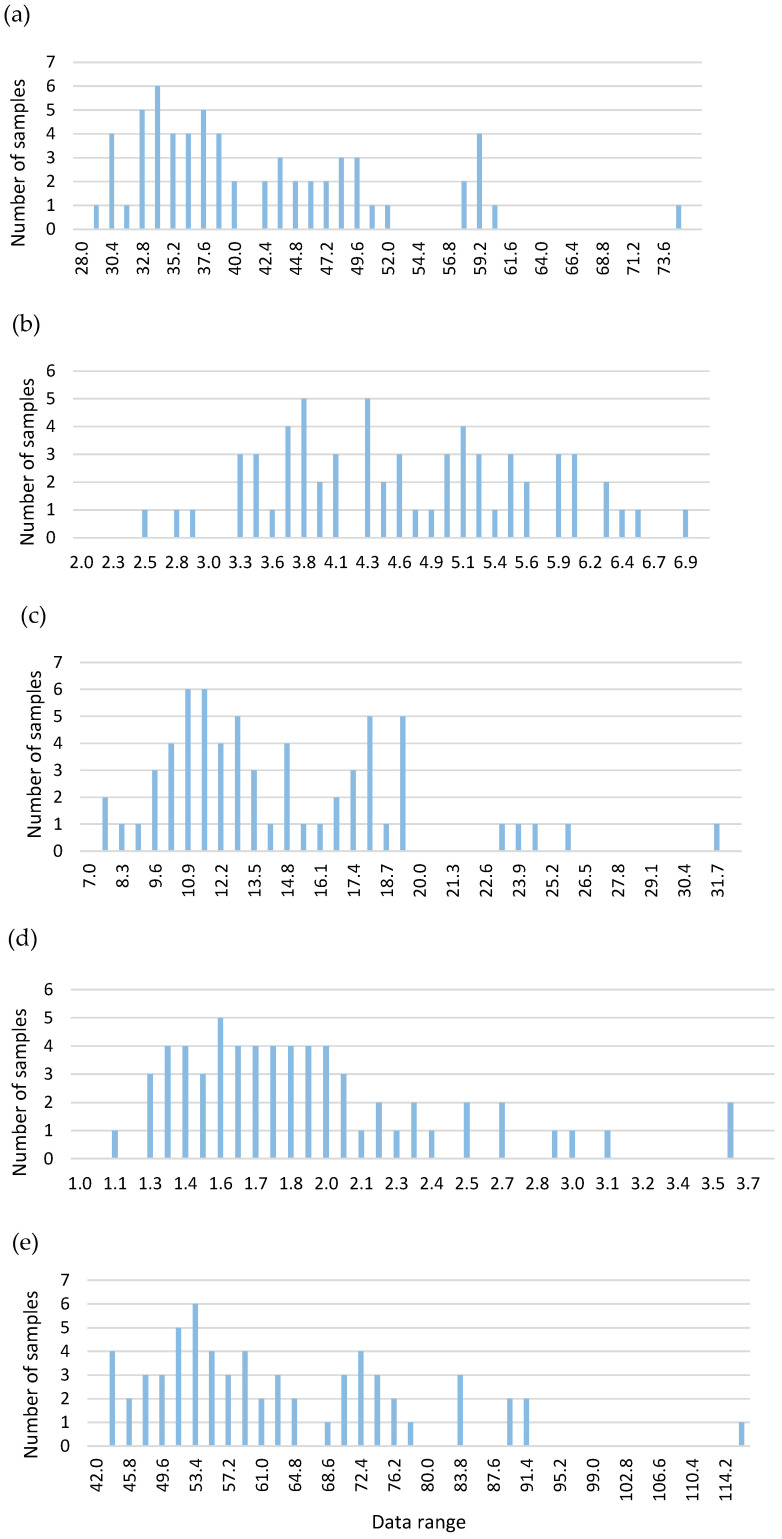
Frequency histograms of the analyzed compounds. (**a**) MA; (**b**) iBA; (**c**) OA; (**d**) UO; (**e**) Total.

For calibration, principal component analysis (PCA) was first carried out with all the spectral values (Figure 4) using the CENTER algorithm and WinISI 4.8 software [15], with a focus on the center of the population. The standard H distance was determined as a variant of the Mahalanobis distance [16]. In this analysis, the dimensionality of the population was reduced with the aim of evaluating the presence of anomalous spectra samples. Thus, the spectral distance of the library could be tested to obtain the global distance to the center of the population (GH). These data were used as indicators if the number of samples used in the calibration was sufficient. Moreover, with WinISI software, the information about the NIR spectra can be improved by minimizing the variations in the physical origins. Particle size, packing pressure, dispersion effects, variations in the light path, and other physical effects can disturb the relationship between the NIRS spectra and the reference values. These errors can be minimized by applying mathematical treatments to the spectra. In general, these chemometric methods are used for the correction of dispersion or scatter.

For this calibration, the following approaches were used:

1. Standard normal variate (SNV) correction was used for the correction of variations in the baseline produced by the particle size and the dispersion. The spectra were transformed to log(1/R). This consisted of subtracting the mean of the spectrum from each original absorbance value and dividing this result by its standard deviation [17].
2. Detrend (DT) correction. The application of this second-grade polynomic function to the absorbance values in log(1/R) and the lambda allows for removal of the linear or quadratic curve in the baseline of the spectra due to the different packing pressures [17].
3. SNV and DT (SNVD) correction. This is a combination of both previous corrections, SNV and DT. It corrects the baseline and removes the differences between the spectra corresponding to samples of a similar chemical composition but with different particle sizes [17].

After defining the pool of samples used, calibration of the model was carried out to predict the concentrations of the chemical components of the samples using their spectral information.

The development of the NIR equation consisted of obtaining an appropriate mathematical relationship between the spectral information and the concentration values of the selected samples. Table 2 shows the results obtained for the calibration of the product. The calibration was carried out with the same mathematical treatment applied to the center of the population.

Table 3 shows the results obtained for the validation of the selected samples.

### 2.5. Oxygen Radical Absorbance Capacity (ORAC) Assay

ORAC assay was carried out following the procedure described by Ou et al. [18] and Hua et al. [19] with modifications.

Sample preparation. In Eppendorf tubes, a 0.1 g sample of fine powder was accurately weighed, and 1.5 mL of acetone/water (50:50, *w*:*v*) extraction solvent was added. The mixture was shaken at 400 rpm in a circular rotatory shaker at room temperature for 1 h. Every 10 min, it was mixed by vortexing. The mixtures were centrifuged at 14,000× *g* for 15 min, and the supernatant was used for the analysis.

Assay. All samples and reagents were dissolved and diluted with phosphate buffer (0.075 M, pH 7.4). For the experimental samples, 5, 10, and 20 µL of supernatants were added to 40 µL phosphate buffer and then mixed with 20 µL of 63 nM sodium fluorescein solution in a clear, 96-well microplate and incubated at 37 °C for 15 min. Then, 140 µL of 18.28 mM 2,2′-azobis(2-amidinopropane) dihydrochloride (AAPH) solution was rapidly added to each well. Also, the fluorescence intensity of each sample was determined without the effect of AAPH in wells because the AAPH solution was replaced by the same volume of phosphate buffer. A calibration curve for the trolox standards, at concentrations of 0, 2.5, 5, 10, 20, and 30 µg/mL, was made.

After the 15 min incubation period and vigorous shaking, the microplate was placed in the multifunctional BioTeK Synergy HR microplate reader (ThermoFisher Scientific Inc., Waltham, MA, USA). The system was set in fluorescence mode, and the fluorescence intensity of each well was read 60 times at 1 min intervals. The fluorescence intensity emitted at 535 nm was read at 37 °C after excitation at 485 nm.

The area under the fluorescence intensity curve for AAPH and AAPH-free wells was used to determine the fluorescence inhibition percentage induced by trolox or by the experimental sample. The ORAC values of the samples were obtained for interpolation against trolox calibration curve.

### 2.6. Statistical Analysis

The results are expressed as the mean ± standard error of the mean (SEM). Linear correlations were determined by least-squares regression analysis. The criterion of significance was *p* < 0.05.

## 3. Results and Discussion

In this work, we describe the development of an NIRS equation that relates the concentrations of pentacyclic triterpenoids to the NIRS spectra of samples made from the leaves and seeds of traditional plants found in a Mediterranean forest. Lentiscus (*Pistacia lentiscus*), wild olive (*Olea europaea*), wine (*Vitis vinifera*), and thyme (*Thymus vulgaris*) were the four plant types used. A total of 63 samples of these four plant types with different compositions and particle sizes were made and used for two analyses. The first analysis was a determination of the compositions of pentacyclic triterpenes in these mixtures. We applied the same techniques of extraction and analysis previously used for olive samples. A typical HPLC-UV–Vis chromatogram of these samples is shown in Figure 1. As shown in this chromatogram, maslinic acid (MA), an isomer of betulinic acid (iBA), oleanolic acid (OA), and uvaol (UO) were identified using the external standard method. The concentration of each compound was determined based on the area of each peak, and the results are shown in Table 1. The chromatogram profiles and positions of the different identified compounds have been previously described for olive leaf samples [2]. The concentrations of the total pentacyclic triterpenoids found in these samples represent 6.19% of their wet weight, with 4.13% MA and 1.42% OA. These are high percentages with respect to the total weights of the samples and validate the procedure used for making this additive.

The second determination was the NIR spectra of the same samples. Figure 2 shows the spectra obtained. After the application of a mathematical approach for the creation of the center, the spectral database became quite homogeneous, showing clear absorption peaks, which could facilitate good calibration. The calibration was carried out using the mathematical treatments SNV +D 1,4,4,1, as previously mentioned. The same mathematical treatment was carried out for all parameters. The statistics obtained from the calibration are shown in Table 2.

To analyze these values, the guide values described by Williams [20] were used. According to this study, when the R2 values are higher than 0.90, an excellent calibration is obtained; when the R2 values are between 0.82 and 0.90, the calibration is good; and when the R2 values are between 0.66 and 0.81, a possible quantitative prediction can be made. When the R2 values are between 0.50 and 0.65, the calibration distinguishes between high and low values. The R2 values obtained in our work are between 0.75 and 0.77, providing the possibility of a quantitative prediction with this methodological application.

For the spectral values, PCA was conducted using WinISI software with the aim of developing a quality control standard of the samples used for the model. The results show that an acceptable calibration was carried out for each of the determined compounds: MA, iBA, OA, UO, and Total. This implies that the model predicted the concentrations of each one.

These results and the models were validated using a comparison of the values obtained for the six samples with the two different analysis methods. These samples were not included in the calibration, and the results are shown in Table 3. A good regression (R2) value and an acceptable relative Sep value were observed for MA, OA, and Total. The iBA and UO statistics show a worse response in the NIRS, probably because their ranges and deviations were smaller.

Moreover, the antioxidant capacity of the plant mixture samples was determined using the ORAC assay. The results obtained are shown in Table 4. Compared with other types of samples, the values found are high [18]. These values demonstrated that these samples have a high capacity to destroy reactive oxygen species, opening the door to the use these plant mixture samples as nature prime matter in feed additives for animal production.

NIRS is being used more frequently for safety inspections and quality assessments of agricultural products [21,22]. It has also been applied to olive oil production as a method to monitor and control olive quality parameters [23] or for the detection of herbicides [24]. NIRS models have been developed to estimate the polyphenolic compound concentration [25] and relative water content [26] in olive leaves and to predict the quality of intact olives [27]. Here, we described another application of this methodology: its use in the quantification of pentacyclic triterpenes, which are bioactive compounds present in Mediterranean plant leaves that have important applications in health. These compounds are highly abundant in olive by-products, such as pruned leaves, and can be used as additives in the production of animal meat. This application would permit the use of these by-products for animal alimentation. This is an important use case at this time, as the availability of raw materials is low. In this sense, our results also demonstrated the high antioxidant capacity of these samples when they were used as additives in animal food.

The method described here has many advantages compared to the traditional method. It can be applied directly to dried powder, avoiding the extraction process and HPLC analysis. With the traditional method, both aspects need 2 or 3 days of laboratory work with specialized equipment and technical personnel. This makes the NIRS assay easier and cheaper. The results obtained with the NIRS assay are equivalent to the traditional method and, moreover, it can be used at the factory level to directly determine the concentrations of MA, iBA, OA, and UO and the total concentrations of pentacyclic triterpenes in food additives at the same time that they are produced. This is another interesting advantage over the traditional method that can improve the practical application of NIRS at an industrial level. At analytical laboratories, the application of this new methodology has been used by some companies as a method to estimate the pentacyclic triterpene concentration in these types of samples.

## 4. Conclusions

Here, we present a novel application of NIRS for the quantification of the total or individual (maslinic acid, oleanolic acid, and uvaol) pentacyclic triterpene concentrations in an additive of animal feed obtained from a Mediterranean plant mixture. The concentration of triterpenes and their NIRS spectra were determined in 63 samples. The antioxidant activity of these samples was also determined. A regression model relating the concentration of these compounds and NIRS spectra was developed. A high correlation value for the calibration and validation sets, along with a high residual prediction deviation value, was obtained. This method can be applied directly to the powder additive, avoiding extraction and HPLC analysis. Moreover, at analytical laboratories, it can be used at the factory level for additive powder fabrication.

## Figures and Tables

**Table 1:** Concentrations of pentacyclic triterpenoids found in plant mixture samples.

	MA	iBA	OA	UO	Total
Minimum	28.89	2.40	10.08	1.10	42.47
Maximum	74.08	6.83	29.75	3.60	114.26
Mean	41.27	4.57	14.19	1.87	61.90
SD	9.59	1.03	2.13	0.54	14.68
Total Samples	63	63	63	63	63

The concentration of each compound is expressed as mg per g of wet weight. MA: maslinic acid; OA: oleanolic acid; UO: uvaol; iBA: isomer of betulinic acid. The results are expressed as the mean. SD: standard deviation.

**Table 2:** Results obtained for the calibration of the products.

	Mean	Minimum	Maximum	R2	SECV
MA	39.85	16.28	63.43	0.75	4.97
iBA	4.59	1.48	7.69	0.77	0.78
OA	13.76	0.90	26.62	0.76	2.49
UO	1.78	0.66	2.89	0.75	0.28
Total	60.35	21.83	98.87	0.77	7.40

The data are the mean of all the experimental values. Abbreviations: R2, R-square; SECV: standard error of cross-validation.

**Table 3:** Validation data.

	MA	iBA	OA	UO	Total
N	6	6	6	6	6
Sep	4.61	0.70	1.32	0.44	2.91
R2	0.90	0.76	0.99	0.54	0.97
BIAS	1.75	0.10	-0.66	0.12	0.73

MA: maslinic acid; iBA: isomer of betulinic acid; OA: oleanolic acid; UO: uvaol. The results are expressed as the mean. N: number of samples; Sep: standard error of prediction; R2: R-square; BIAS: systematic error in the prediction.

**Table 4:** Oxygen radical absorbance capacity (ORAC) value for the plant mixture samples.

mg trolox equivalents/g of wet sample	9877.62 ± 466.5 (9)
mmol trolox equivalents/100 g of wet sample	3944.90 ± 186.3 (9)

Results are expressed as the mean ± standard error of the mean, with the number of determinations between parentheses.

## Data Availability

The original contributions presented in this study are included in the article; further inquiries can be directed to the corresponding author.
